# Deep Structural Characterization of Protein-Bound Lipids via Native MS and Ultraviolet Photodissociation

**DOI:** 10.1021/acs.analchem.5c03691

**Published:** 2025-08-28

**Authors:** Carla Kirschbaum, Jack L. Bennett, Carol V. Robinson

**Affiliations:** † Kavli Institute for Nanoscience Discovery, 6396 University of Oxford, Oxford OX1 3QU, United Kingdom; ‡ Department of Chemistry, University of Oxford, Oxford OX1 3QU, United Kingdom

## Abstract

Protein–lipid interactions are critical for maintaining membrane protein structure and regulating diverse protein functions. Native mass spectrometry (MS) has emerged as a powerful technique for the direct observation and characterization of protein–lipid complexes. However, intact mass measurements alone cannot resolve important structural details such as the identity of lipid acyl chains and their modifications. To fully characterize protein-bound lipids, we present a multistage native MS method that leverages ultraviolet photodissociation to elucidate the precise molecular composition of heterogeneous protein–lipid assemblies. We demonstrate the utility of this approach for both soluble and membrane proteins. First, we comprehensively define the endogenous lipids bound to the bacterial transporter MlaC, distinguishing between unsaturated and cyclopropane lipids, and localizing acyl chains and their modifications. Next, we characterize and quantify phospholipids associated with the bacterial membrane protein AqpZ and show that the approach can be extended to more complex cardiolipins containing four lipid chains. Together, our workflow provides detailed structural insights into protein–lipid interactions and offers a path toward uncovering protein-specific metabolic regulation that is not accessible through classical lipidomics workflows.

## Graphical abstract

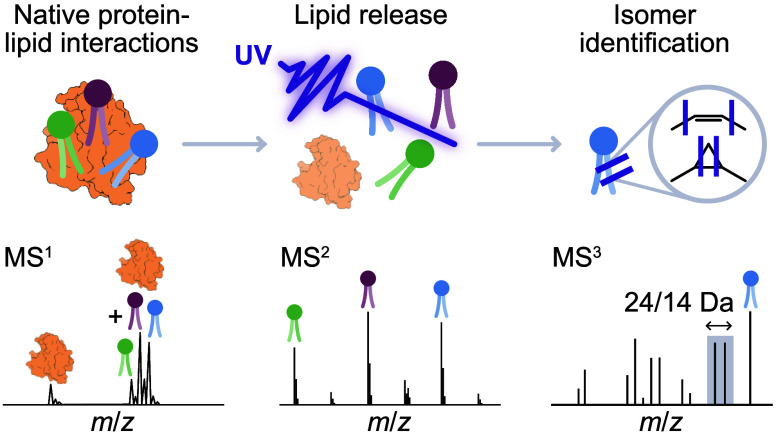

## Introduction

Protein–lipid interactions play critical roles in shaping the structure and function of integral membrane proteins and membrane-associated proteins.^1^ Membrane proteins are often laterally sorted to specific membrane regions, where they can create a unique nanoenvironment by recruiting specific lipids.^1,2^ The lipid environment influences protein behavior on multiple levels, ranging from specific, high affinity interactions to broader modulation of bulk membrane properties.^3^ These interactions can influence protein conformation and mediate oligomerization, both of which are intimately linked with protein function.^4,5^ However, determining the role of individual lipids, whether tightly bound or in the bulk, remains a major challenge for current analytical techniques.

The diversity of lipids within a single cell is immense.^6^ As secondary gene products, lipids are not genetically encoded but arise from metabolic processes shaped by the environment. In eukaryotic cells, membrane lipid composition is fine-tuned by lipid transfer proteins, which transport specific lipids from their site of synthesis to the target organellar or plasma membranes.^7,8^ Such directed trafficking creates unique lipid compositions in different organelles^9^ by leveraging the high selectivity of lipid transfer proteins and exploiting lipid gradients as a driving force.^10^ Lipid profiles vary between organelles and cell types, and are dynamically reshaped by factors such as diet, metabolism, and disease.^6^ In particular, lipid metabolism is reprogrammed in cancer cells,^11^ including the exploitation of alternative lipid synthesis routes which generate atypical lipid isomers.^12^

Classical approaches to studying protein–lipid interactions often involve *in vitro* assays employing artificial bilayers or liposomes, which are based on the immobilization of one interaction partner.^13^ Alternatively, cell-based lipid perturbation assays are used to measure the phenotypic outcome of modified lipid metabolism.^13^ While informative, these methods generally lack the resolution needed to characterize lipid composition and structure. Native mass spectrometry (MS) has emerged as a powerful technique for obtaining such deep structural insights.^14,15^ Intact protein–lipid complexes can be directly observed in the gas phase, and systematically fragmented to identify the ensemble of bound lipids with exceptional structural precision. Beyond exact mass measurements of the released lipids, multistage fragmentation enables unambiguous identification of different lipid classes based on their characteristic fragmentation patterns.^14,15^

Phospholipids exhibit multiple levels of structural complexity: lipid class (defined by the headgroup), fatty acid identities, their respective positions on the glycerol backbone (*sn*-position), and position and configuration of C=C bonds or other modifications within the fatty acid chains.^16^ Conventional MS-based structural analysis, most commonly using low-energy collisional activation, only yields information up to the second to third level, *i*.*e*., lipid class, acyl chain identities, and restricted information on acyl chain positions.^17^ However, structural details such as acyl chain modifications, typically remain unresolved, despite their importance in metabolic diseases.

To determine the exact structure of phospholipids, a range of advanced tandem MS-based techniques have been developed.^18–20^ Many of these rely on chemical modification of lipids either prior to MS, where collisional activation of the derivatized analytes yields diagnostic fragments, or within the mass spectrometer via ion–ion or ion–molecule reactions. As an alternative, nonergodic activation methods, such as ultraviolet photodissociation (UVPD) have shown promise for lipid structural elucidation.^21^ UVPD cleaves C–C bonds in unmodified lipid ions, producing complex yet informative fragment spectra across diverse lipid classes.

Initial studies using 193 nm UVPD demonstrated that C=C bonds in unsaturated lipids yield diagnostic fragment ion pairs spaced by 24 Da, enabling localization of double bonds and discrimination of isomers (Scheme 1).^22^ Similarly, cyclopropane modifications in bacterial lipids can be identified and localized based on fragment ion pairs spaced by 14 Da (Scheme 1).^23^ UVPD has also been used to localize other lipid modifications, such as hydroxylation and alkyl chain branching.^24^

**Scheme 1:**
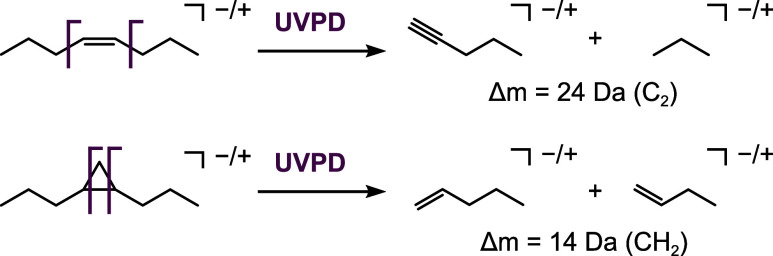
UVPD of Unsaturated and Cyclopropane Lipids^a^ ^22,23^

a Photodissociation of C–C bonds left and right to C=C bonds yields a fragment ion pair separated by 24 Da. Cleavage of C–C bonds flanking cyclopropane rings results in two fragment ions spaced by 14 Da. Putative fragment structures are shown.^22,23^

Moreover, a hybrid collisional activation/UVPD scheme has been developed to determine acyl chain positions (*sn*-1/*sn*-2) in glycerolipids.^25^ It is based on the formation of dioxolane rings upon collisional activation of alkali metal-adducted glycerolipids.^25–27^ The metal-adducted dioxolanes yield unique cross-ring fragments for *sn*-isomers in the subsequent UVPD stage (Scheme 2).^25^

**Scheme 2:**
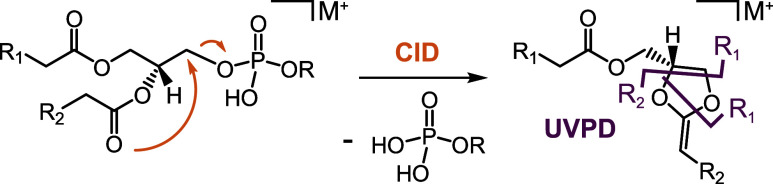
Hybrid Collisional Activation/UVPD Scheme for Identification of Acyl Chain Positions.^a^ ^a^Collisional activation of phospholipid metal adducts (e.g., Na^+^) followed by UVPD of the resulting dioxolane fragment yields three unique cross-ring fragments that allow to infer acyl chain positions (*sn*-1 and *sn*-2).

By integrating UVPD into structural lipidomics workflows, cell-wide changes in the lipidome can be registered as a function of metabolic state.^28^ However, changes in the lipidome on the cellular level can have different impacts on the nanoscopic level by influencing functional molecular hubs around proteins. Proteins recruit specific, sometimes low-abundant, lipids that are not easily picked up in a bulk lipidomics analysis but are crucial for oligomer stabilization or protein function.^4,29,30^ To study lipids that are tightly bound to proteins or within their immediate nanoenvironment, we need to develop a workflow that focuses on individual protein–lipid complexes.

UVPD is particularly attractive for characterizing protein-bound lipids because it does not involve chemical modifications that could affect native protein–lipid interactions. To date, however, UVPD has only been applied on isolated lipids, or lipids released nonspecifically from protein samples. A recent study showcased UVPD of lipids released from a protein, demonstrating the potential of this approach.^31^ However, without isolating the intact protein–lipid complex in the mass spectrometer, contaminants from detergent micelles or the bulk lipid phase, could obscure direct protein–lipid interactions.

Here, we present a multistage native MS workflow that enables deep structural characterization of phospholipids released from intact protein–lipid complexes. Using a high-mass-range linear ion trap, we systematically isolate and activate protein–lipid complexes. The lipids are released and identified using a combination of collision-based activation and UVPD. A key feature of our approach is the preservation of protein–lipid interactions by isolation of intact protein–lipid complexes, ensuring that only specifically bound lipids are analyzed. This approach enables unprecedented structural detail of protein-associated lipids and opens new avenues for studying the role of specific lipids in modulating membrane protein function.

## Materials and Methods

### Lipid Standards

POPC (PC 16:0/18:1­(9*Z*)) IsoPure, OPPC (PC 18:1­(9*Z*)/16:0) IsoPure, DOPG (PG 18:1­(9*Z*)/18:1­(9*Z*)), DOPE (PE 18:1­(9*Z*)/18:1­(9*Z*)), PE 16:0/17:1­(9cy), and CDL 18:1­(9*Z*) were obtained from Avanti Research, dissolved in chloroform, and diluted in methanol to a final concentration of 10 μM for mass spectrometry. *E. coli* lipid extract was purchased from Avanti Research, dried overnight in a centrifugal evaporator, and resuspended by vortexing in 200 mM ammonium acetate (pH 7.0) containing 2×CMC C8E4 to an estimated total lipid concentration of 5 mM (assuming an average molecular weight of 750 g/mol). Small unilamellar vesicles were generated by sonication for 30 min.

### Protein Expression

MlaC was expressed in *E. coli* BL21­(DE3) cells as reported previously without modifications.^32^ Briefly, full-length MlaC was expressed with a hexahistidine tag and TEV cleavage site following the N-terminal signal sequence. Cells were grown at 37 °C in Luria broth under kanamycin selection (50 μg/mL) to an OD_600_ of 0.5 before induction with IPTG (0.8 mM) and overnight expression at 16 °C. Cells were lyzed in a microfluidizer, and MlaC was purified by Ni-NTA affinity column chromatography. After dialysis and cleavage of the His tag with TEV protease overnight, MlaC was further purified by reverse Ni-NTA affinity column chromatography and size exclusion chromatography. The final buffer contained 50 mM Tris pH 8.0, 150 mM NaCl. MlaC was buffer-exchanged into 200 mM ammonium acetate (pH 7.0) for native MS.

AqpZ was expressed in *E. coli* BL21­(DE3) cells with a TEV protease-cleavable C-terminal GFP-His tag, as reported previously.^33^ Briefly, cells were grown under ampicillin selection (100 μg/mL) to an OD_600_ of 0.5, induced with IPTG (0.5 mM), and grown for 4 h at 37 °C. Cells were lyzed in a microfluidizer, and membranes were isolated by ultracentrifugation. The membranes were solubilized with 1% DDM for 1 h. AqpZ was purified by Ni-NTA affinity column chromatography, followed by cleavage of the GFP tag overnight using TEV protease, reverse Ni-NTA affinity column chromatography, and size exclusion chromatography. The final buffer contained 50 mM Tris pH 8.0, 150 mM NaCl, 10% glycerol, 2×CMC DDM. For native MS, AqpZ was buffer-exchanged into 200 mM ammonium acetate (pH 7.0) containing 2×CMC C8E4.

### Native MS

Mass spectrometry measurements were performed on an Orbitrap Eclipse Tribrid mass spectrometer equipped with a 213 nm UV laser (Thermo Fisher Scientific). Protein–lipid complexes were ionized by nanoelectrospray ionization using gold-coated borosilicate capillaries pulled and coated in house. An optimal irradiation time of 500–1000 ms was determined by UVPD of lipid standards in positive and negative ion modes.

Native mass spectra of MlaC were obtained in Peptide mode without source activation. Phospholipids were released from the MlaC−lipid complex using ion trap isolation (pos. mode: 9+, *m*/*z* 2508 ± 25; neg. mode: 8–, *m*/*z* 2816 ± 25) and ion trap CID (pos. mode: 25% NCE, 50 ms, *q* = 0.12; neg. mode: 20% NCE, 50 ms, *q* = 0.12). MS^2^ spectra were recorded using the Orbitrap at a resolution of 500,000@*m*/*z* 200. MS^3^ and MS^4^ spectra involving UVPD were recorded in the ion trap (injection time: 100 ms, AGC target 100–1000%). Each UVPD spectrum was averaged for 300–600 scans for MS^3^ spectra and 500–999 scans for MS^4^ spectra, depending on the precursor ion abundance. For lipid class and acyl chain identification, HCD spectra of all released lipids were recorded in positive and negative ion mode, respectively. The monoisotopic peak of intact lipid precursor ions was isolated using an isolation window of 1.5 Th, and fragmented using HCD (25–30% NCE).

Detergent-purified AqpZ (ca. 10 μM) was incubated in solution with small unilamellar vesicles of *E. coli* lipid extract (ca. 500 μM) in 200 mM ammonium acetate (pH 7.0) containing 2×CMC C8E4. Native mass spectra with resolved lipid-bound states were obtained in Intact Protein mode using 150 V in-source activation (compensation factor = 0.1). AqpZ–lipid complexes were dissociated in negative ion mode using ion trap isolation (17–, *m*/*z* 6000 ± 150) and HCD (15–20% NCE). Multistage native MS on released lipids was performed as described above. For relative quantification of lipids bound to AqpZ, the intensities of released lipids were compared to the mass spectrum of the lipid extract dissolved in methanol (10 μM) obtained in triplicate in Small Molecule mode.

### UVPD Spectra Analysis

UVPD spectra were analyzed using integrated custom code. Briefly, the algorithm generates a list of putative lipids (sum composition) based on intact lipid masses in the MS^2^ spectrum after internal linear recalibration. Lipid class, adduct type and acyl chain length are derived from HCD spectra of the released lipids in positive and negative ion modes. The lipid class and adduct type are determined in positive mode based on diagnostic fragmentation (PE­(H^+^): neutral loss of 141 Da; PE­(Na^+^): neutral loss of 43 and 141 Da; PG­(Na^+^): neutral loss of 172 Da and 194 Da). In negative mode, the acyl chain identities are determined based on carboxylate ion pairs that sum up to the correct lipid mass. Putative *sn*-positions are assigned if the fragment intensity ratio of carboxylate ion pairs is at least 2.5:1 (*sn*-2:*sn*-1). In the absence of HCD spectra, acyl chain identities can also be derived based on neutral acyl chain loss in the UVPD spectra. Based on the knowledge of lipid class, adduct type, and acyl chain identities, a library of possible UVPD fragment ion pairs is generated for double bonds and cyclopropane modifications. The UVPD spectra of protonated and deprotonated lipid ions are scanned for fragments spaced by 14 or 24 Da and matched with fragment ion pair masses in the library for the respective lipid.

### Relative Quantification of *sn*-Isomers

For quantification of *sn*-isomers based on MS^4^ spectra, a calibration curve was generated by mixing different ratios of POPC and OPPC lipid standards. The intensity ratios of the main peaks measured for each isomer (*m*/*z* 319 and 345) were determined at each concentration in triplicate. Differences in stock solution concentrations were accounted for by a postmeasurement scaling factor. In accordance with the shorthand notation for lipids,^16,34^ phospholipids are reported with a slash if the acyl chain positions of the major isomer were clearly identified. Underscores are used in cases where the acyl chain positions could not be determined or mixtures of *sn*-isomers were present.

## Results and Discussion

### Overview of the Workflow

We present a workflow combining native MS with UVPD for the deep structural characterization of protein-bound lipids. It combines sequential ion trap isolation with multimodal ion activation to release and identify lipids from intact protein–lipid complexes (Figure 1). The workflow begins with acquiring a native mass spectrum of the protein of interest under conditions which preserve native protein–lipid interactions. A native mass spectrum of protein–lipid complexes typically contains a fraction of *apo* protein and several lipid-bound states. For soluble proteins with a defined lipid binding pocket, the protein:lipid stoichiometry is generally fixed, *e*.*g*., 1:1. In contrast, membrane proteins often bind many lipids across their hydrophobic surfaces, resulting in a wide range of stoichiometries in the native mass spectrum. As we have previously demonstrated, binding of individual lipids can be quantified from the MS^1^ envelope using MS^2^-guided curve fitting, provided that individual lipid-bound states are partially resolved in the MS^1^ spectrum.^32^

**Figure 1:**
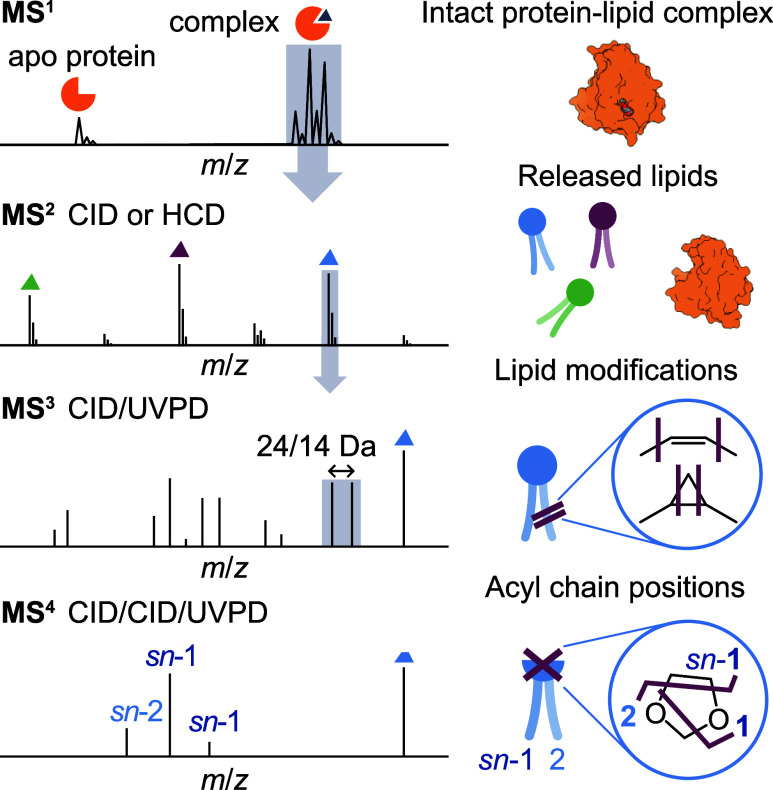
Overview of the workflow combining native MS with UVPD to analyze structural details of protein-bound lipids. Intact protein–lipid complexes are isolated in the gas phase and subjected to collisional activation to release bound lipids. Each lipid is individually isolated and subjected to UVPD to reveal acyl chain modifications including unsaturations or cyclopropanation. The respective positions of the two acyl chains can be (optionally) identified in an MS^4^ experiment using a CID/CID/UVPD sequence.

At the MS^2^ stage, lipids are released from the protein by isolating an ensemble of protein–lipid complexes and applying collision-induced dissociation (CID) or higher-energy collisional dissociation (HCD). This process liberates isolated lipids either as neutral molecules or charged ions. The latter typically appear as singly charged ions in the low *m*/*z* range, whereas charge-reduced protein ions are observed in the higher *m*/*z* region. The released lipids can then be further characterized by ion-trap isolation and collisional activation (MS^3^) to reveal the lipid class and acyl chain composition.

To obtain more detailed information on acyl chain structure and modifications, we apply 213 nm UVPD to intact phospholipids at the MS^3^ stage. UV irradiation yields informative fragment ions that allow us to identify and localize chain modifications such as C=C bonds or cyclopropane rings (*cf*. Scheme 1). To determine acyl chain positions on the glycerol backbone, sodiated lipids are fragmented using CID to generate dioxolane fragments. The latter yield *sn*-specific cross-ring fragments upon UVPD in an MS^4^ experiment (*cf*. Scheme 2).

Together, this workflow enables the precise characterization of lipids bound to proteins, including lipid class, acyl chain identity, acyl chain position and acyl chain modifications. In the following sections, we will discuss the applications of this approach to characterize the lipid cargo of a bacterial lipid transporter and a membrane protein.

### MlaC–A Bacterial Lipid Transporter

To evaluate the UVPD-based workflow we first investigated MlaC, a soluble lipid transporter that shuttles phospholipids between the inner and outer membrane of Gram-negative bacteria.^35^ The native mass spectrum of MlaC expressed in *E. coli* featured three major charge states in positive ion mode (8–10+) and contained peaks corresponding to diverse lipid adducts with an apparent 1:1 binding stoichiometry (Figure 2A). We released these copurified lipids from the MlaC–lipid complex in both positive and negative ion modes using CID (9+ or 8– charge state assigned to the protein–lipid complexes). We detected protonated and sodiated lipids as well as deprotonated lipids in positive and negative ion modes respectively (Figure 2B). We assigned the lipid class and sum composition based on CID/HCD MS^3^ spectra, as reported previously (Figure S7).^32^ All lipids could be assigned to either phosphatidylethanolamine (PE) or phosphatidylglycerol (PG), the two most abundant phospholipid classes in *E. coli*. Using collisional activation only, we could not distinguish between unsaturated and cyclopropane lipids, nor localize the modifications along the lipid chains.

**Figure 2:**
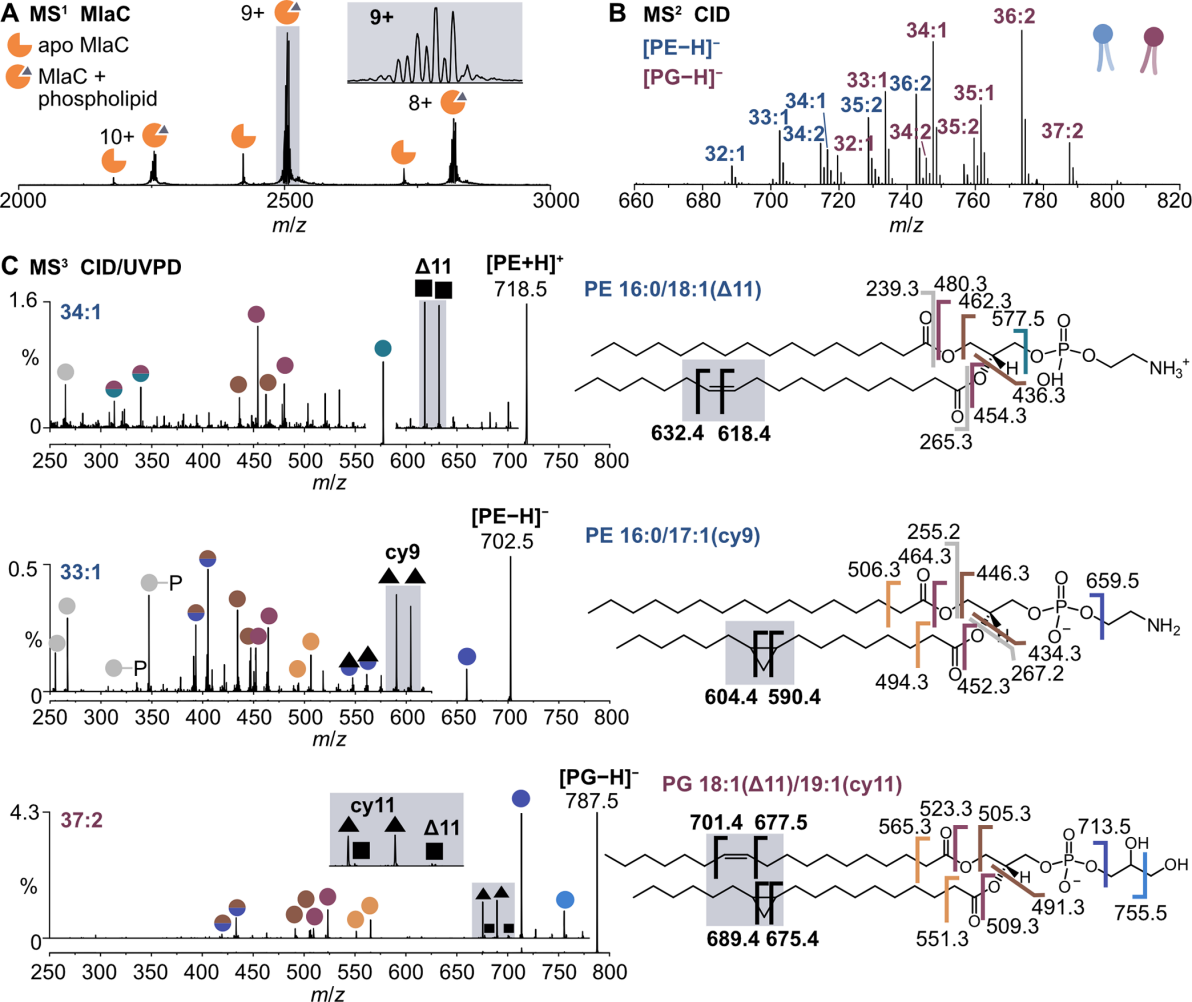
Characterization of lipids transported by the bacterial lipid transfer protein MlaC. (A) Native mass spectrum of MlaC copurified with phospholipids from *E. coli*. (B) Isolation and CID of the protein–lipid complex (9+, *m*/*z* 2508 ± 25) releases PE and PG with varying acyl chain compositions. (C) UVPD of the released lipids reveals the position of C=C bonds (squares) and cyclopropane modifications (triangles). Other fragments result from cleavage of acyl chains or the headgroup (colored circles). Dual-colored circles indicate fragments that arise from cleavage at two sites.

To overcome this limitation and characterize acyl chain modifications in all phospholipids bound to MlaC, we applied CID/UVPD MS^3^ measurements, in positive and negative ion modes. To guide assignment of the resulting spectra, we also measured UVPD MS^2^ spectra of synthetic lipid standards (DOPE, PE 16:0/17:1­(cy9) and DOPG) in positive and negative ion modes (Figures S1–S3). While DOPG and cyclopropane PE yielded the expected fragment ion pairs for C=C bonds and cyclopropane rings, we found that both protonated and sodiated unsaturated PE ions yielded diagnostic fragment ions spaced by 14 Da instead of the 24 Da expected for C=C bonds. This aberrant fragmentation behavior of unsaturated PE has been reported previously and complicates the analysis;^36^ because successive cleavages along alkyl chains would also generate ion series separated by 14 Da, fragment ion pairs spaced by 14 Da are less diagnostic than 24 Da spacing. Nonetheless, the most abundant fragment ion pair is diagnostic of the most abundant double bond isomer. Furthermore, C=C bonds and cyclopropane modifications in PE lipids can be distinguished from each other despite identical fragment ion pair spacing in positive ion mode, because their respective fragment structures differ by two hydrogens, leading to a mass shift of 2 Da (Figure S5).

For protonated DOPE, we further observed an unexpected fragment ion pair spaced by 14 Da, which was shifted by 98 Da relative to the double bond-diagnostic fragment ion pair. High-resolution Orbitrap UVPD confirmed that the mass difference corresponded to the loss of H_3_PO_4_ (97.98 Da), suggesting complex gas-phase reactions of protonated unsaturated PE upon UVPD. We took this finding into consideration for the analysis of unknown phospholipids to prevent mistaken assignment of the fragment ion pair as another double bond shifted by seven methylene groups (theoretical mass shift 98.11).

We further used the synthetic lipids to determine optimal irradiation times for each lipid class and obtained good results across all three lipid standards with 500–1000 ms irradiation. When evaluating fragment intensities in positive and negative ion modes for different adduct types, we found that cyclopropane modifications in PE were best detected by UVPD of deprotonated precursor ions, whereas protonated precursors yielded the most abundant diagnostic fragments for C=C bonds. Double bonds in DOPG were best identified by fragmentation of deprotonated ions. Optimal ion polarities and ion types for localizing modifications in different bacterial phospholipids are summarized in Table S3. We used the combined insights gained from fragmentation of synthetic lipid standards to develop an algorithm to interpret the UVPD spectra of PE and PG lipids released from MlaC. It links MS^2^ spectra of lipids released from proteins to MS^3^ spectra obtained using collisional activation or UVPD to derive the lipid class, acyl chain composition, and chain modifications in an integrated manner (Figure S6).

The UVPD spectra of phospholipids copurified with MlaC showed an abundance of informative fragments for structural assignment (Figure 2C). The UVPD spectrum of protonated PE 34:1 contained several peaks derived from cleavage of the two fatty acids (FA), enabling us to confirm their composition (FA 16:0 and FA 18:1). The spectrum was dominated by a fragment ion pair spaced by 14 Da at *m*/*z* 618.4 and 632.4, which corresponded to a C=C bond at the Δ11 position in the 18:1 chain. The cyclopropane modification in PE 33:1 was more readily detected by UVPD in negative ion mode. The detected fragment ion pair spaced by 14 Da at *m*/*z* 590.4 and 604.4 was consistent with a cyclopropane ring at position 9 in the 17:1 chain. For PE carrying both a C=C bond and cyclopropane modification, such as PE 37:2 (18:1/19:1), reliable localization of both modifications is optimally achieved by fragmentation in both ion modes (Figure S5).

In the case of PG, both types of lipid modification could be detected by irradiating deprotonated precursor ions with 213 nm UV photons (Figure 2C). As shown for PG 37:2, which carries a C=C bond in one chain and a cyclopropane ring in the other chain, cyclopropane modifications yielded more abundant fragment ions (*m*/*z* 675.4, 689.4) than C=C bonds (*m*/*z* 677.5, 701.4). A drawback of irradiating intact phospholipids with UV photons was that we could not confirm which of the two modifications was contained within each chain. However, based on the biochemistry of bacterial lipids, the cyclopropane modification must be in the odd chain (FA 19:1), as cyclopropane lipids are synthesized by methylation of double bonds in even-chain fatty acids.^37^ Based on this prior knowledge, we pinpointed the double bond to position Δ11 in the 18:1 chain and the cyclopropane ring to position 11 in the 19:1 chain. Using this approach, we assigned modifications in all phospholipids copurified with MlaC. Altogether, we identified four major fatty acids in the phospholipids, 16:1 (Δ9), 17:1 (cy9), 18:1 (Δ11), and 19:1 (cy11). All UVPD spectra and assignments are shown in Figure S8 and Table S5.

To determine the relative positions of the acyl chains at the glycerol backbone (*sn*-1/*sn*-2), we performed CID/CID/UVPD MS^4^ experiments on sodiated phospholipids released from MlaC. Upon CID, sodiated PE and PG underwent neutral headgroup loss, forming protonated and sodiated dioxolane fragments. We reisolated the sodiated dioxolane fragment and induced cross-ring cleavage by UV irradiation. This reaction afforded two cross-ring fragments carrying the fatty acyl at the *sn*-1 position, and one fragment carrying the other fatty acyl chain.

In the MS^4^ spectra of PE and PG 34:1, released from MlaC, the most intense peak (*m*/*z* 319) corresponded to one of the *sn*-1-specific fragments carrying palmitic acid (FA 16:0) (Figure 3A). The major *sn*-isomer was inferred to be 16:0/18:1 (*sn*-1/*sn*-2) in both cases. However, we also detected the fragment ion mass consistent with a fragment carrying FA 18:1 at the *sn*-1-position (*m*/*z* 345). To quantify the *sn*-isomer ratio, we obtained CID/UVPD MS^3^ spectra of the lipid standards PC 16:0/18:1 (POPC) and PC 18:1/16:0 (OPPC) at different concentration ratios (Figure 3B). Upon CID, the two standards formed identical dioxolane fragments as PE and PG through neutral headgroup loss. The intensity ratio of the main fragments generated by UVPD, I_319_/(*I*_345_ + *I*_319_), scaled linearly with the POPC/OPPC ratio, increasing from 2% to 97% as the POPC ratio was increased from 0% to 100%. The intensity ratio measured in the MS^4^ spectra of PE and PG 34:1 released from MlaC was 0.9. Based on our calibration, this corresponded to a mix of isomers containing ca. 95% 16:0/18:1 and 5% 18:1/16:0 bound to MlaC.

**Figure 3:**
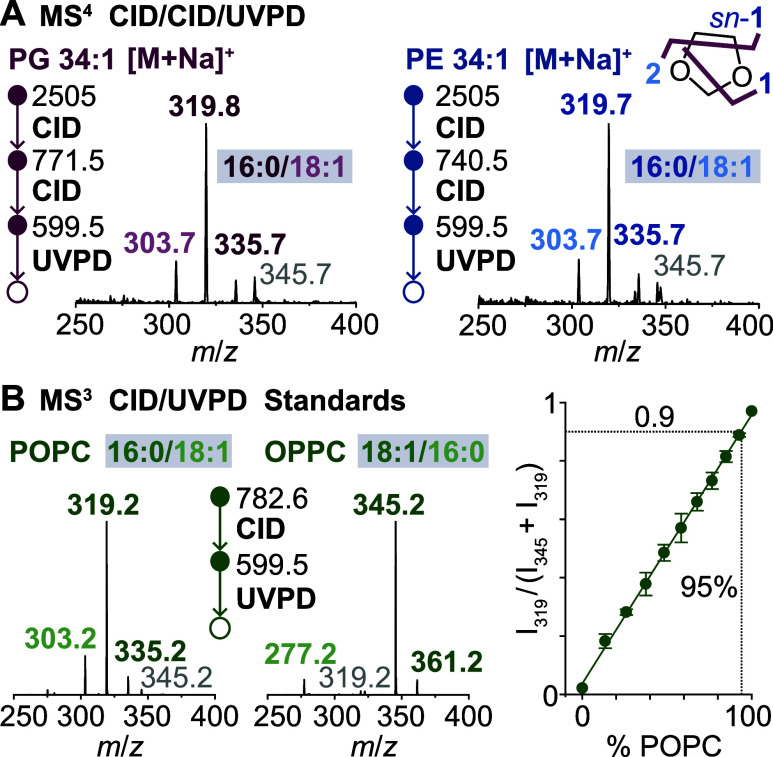
Determination of acyl chain positions by a combination of collisional activation and UVPD. (A) MS^4^ spectra of PG and PE 34:1 bound to MlaC. The acyl chain composition of the main isomer (ca. 95%) is 16:0/18:1 (*sn*-1/*sn*-2) (B) MS^3^ spectra of the reference standards POPC and OPPC were used for relative quantification of *sn*-isomers by measuring the intensity ratios of the two most abundant fragments (*m*/*z* 319 and 345) at varying concentration ratios.

Using the MS^4^ approach, we were able to assign the major *sn*-isomer for all lipids released from MlaC (Table S4 and Figure S9). Together with the data obtained from the UVPD spectra of intact phospholipids, we achieved full characterization of all lipids transported by MlaC in *E. coli*, except for double bond configuration. The ability to gain such deep structural information about individual lipids present in protein–lipid complexes to the best of our knowledge has not been demonstrated with any other technique to date. When comparing the identified lipids to the bulk lipid extract, we could not identify any enrichment of acyl chain or *sn*-isomers. Nonetheless, this approach would allow to pick up lipid selectivities if a protein did enrich specific isomers. To explore whether the approach can be extended beyond soluble lipid binding proteins, we next tested the workflow on a bacterial membrane protein.

### AqpZ–A Bacterial Water Channel

Aquaporin Z (AqpZ) is a bacterial water channel consisting of four identical subunits that span the entire membrane. We expressed GFP-fused AqpZ in *E. coli* and found that the amount of endogenously bound lipids after detergent extraction and affinity purification was not sufficient for the multistage native MS workflow. We therefore opted for an *in vitro* binding assay to determine which lipids preferably bind to the membrane protein.

First, to investigate the conditions under which bacterial phospholipids can be bound and released, we incubated AqpZ individually with the lipid standards DOPE, DOPG, and cardiolipin (CDL, all 18:1). The resulting native mass spectra confirmed that all three lipid classes bind to the membrane protein (Figure S10). Contrary to MlaC, we could only detect released lipids in negative ion mode; positively charged protein–lipid complexes underwent neutral lipid loss upon collisional activation, whereas the protein retained the charge. In general, we have observed that the ability to release charged phospholipids from protein–lipid complexes is highly system-dependent and depends not only on the protein but also the lipid class and its propensity to ionize in the respective ion mode. Release of cardiolipin from AqpZ required higher collision energies than needed for the release of DOPG and DOPE (20% vs 15% NCE), which concomitantly led to some fragmentation of AqpZ (ca. 25%). We isolated the released lipids and found that the ion abundance was sufficient to obtain UVPD spectra which confirmed the known double bond position for all three lipid standards (Figure S10).

We then incubated AqpZ with a complete *E. coli* lipid extract. The resulting native mass spectrum showed multiple lipid-bound states (Figure 4A). We isolated charge state 17– (inset in Figure 4A) and applied HCD to the protein–lipid complexes. The released lipids included all phospholipid classes present in *E. coli*: PE, PG, and CDL (Figure 4B). While phospholipids with longer chains are marginally enriched in the lipid ensemble released from AqpZ, compared to those in the lipid extract, we observe overall little selectivity of AqpZ for specific lipids (Figure S11).

**Figure 4:**
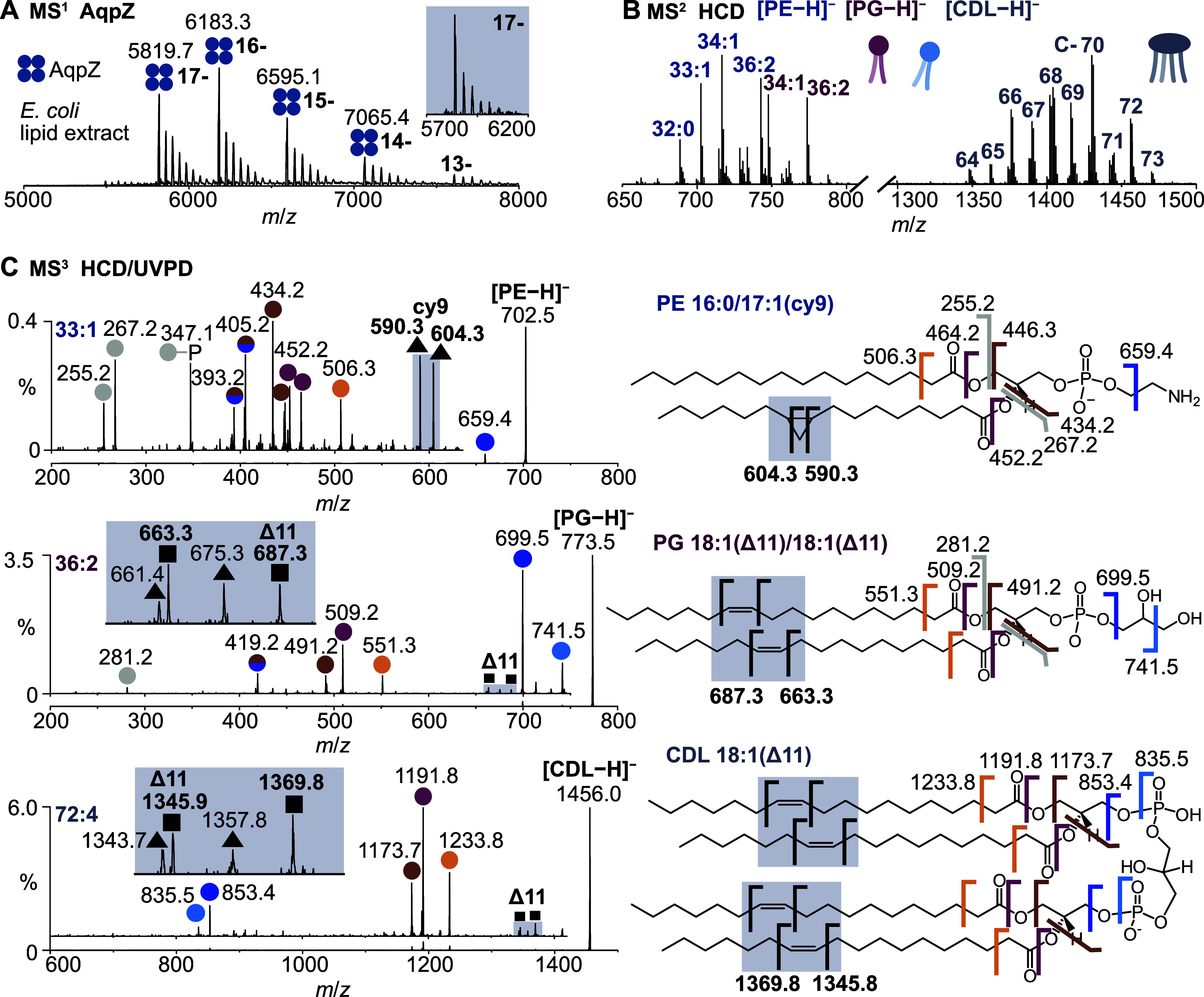
Characterization of lipids bound to AqpZ after incubation with an *E. coli* lipid extract. (A) Native mass spectrum of AqpZ with lipid-bound states. The inset shows the isolation window for lipid release (17–, *m*/*z* 6000 ± 150). (B) Collisional activation of the protein–lipid complex in negative ion mode reveals that AqpZ binds PE, PG, and CDL. (C) UVPD spectra of the released lipids allow localization of C=C bonds (squares) and cyclopropane modifications (triangles). Other fragments result from cleavage of acyl chains or the headgroup (colored circles). Dual-colored circles designate fragments that arise from cleavage at two sites.

To explore the abilities of UVPD to identify lipids released from AqpZ in an MS^3^ experiment, we first selected PE 33:1, which we could compare to the synthetic standard and to the MS^3^ spectrum obtained from MlaC (*cf*. Figure 2C). UVPD yielded a fragment spectrum of similar quality as PE 33:1 released from MlaC. The fragment ions matched with the expected fragment masses (Figure 4C) and confirmed a cyclopropane modification at position 9 in the 17:1 chain.

Next, we applied UVPD to PG 36:2, which has the same molecular sum composition as the DOPG lipid standard. HCD of the released lipid revealed that the prevalent fatty acid composition was two FA 18:1. A low-abundant isomer, containing FA 17:1 and 19:1, was also present (Figure S12). The predominant fragments in the UVPD spectrum of PG 36:2 were identical to the fragments of DOPG; however, the fragment ions indicative of chain modifications differed. Compared to DOPG, which carries a Δ9 double bond in both chains, the double bond-specific fragments of PG 36:2 released from AqpZ were shifted by 28 Da (*m*/*z* 663.3, 687.3), indicative of C=C bonds at position Δ11. Furthermore, we detected a fragment ion pair spaced by 14 Da (*m*/*z* 661.3, 675.3), consistent with a cyclopropane modification in the minor 17:1_19:1 isomer. Even though that isomer is less abundant, the observation is consistent with our previous results which showed that cyclopropane modifications yield more abundant fragments than C=C bonds for PG containing both modifications (*cf*. Figure 2C).

Finally, we explored if the UVPD approach was also amenable to the structural analysis of cardiolipin, a more complex phospholipid that contains four acyl chains. We chose to fragment CDL 72:4, because it has the same molecular sum formula as the CDL (18:1) lipid standard. Using an HCD/HCD sequence, we found that the main fatty acid in CDL 72:4 released from AqpZ was FA 18:1. Low-abundant fragment ions for FA 17:1 and 19:1 were also present (Figure S12). The most abundant CDL isomer thus contains four FA 18:1, whereas minor isomers exist in which FA 17:1 and 19:1 replace two FA 18:1. Accordingly, we detected a fragment ion pair spaced by 24 Da (*m*/*z* 1345.8, 1369.8) in the UVPD spectrum, which corresponds to a C=C bond at position Δ11 in the 18:1 chains. As previously observed for PG 36:2, a second fragment ion pair spaced by 14 Da (*m*/*z* 1343.7, 1357.8) reflected the presence of other isomers containing FA 17:1 and 19:1 with cyclopropane modifications at position 9 and 11, respectively. Other assignments coincide with headgroup-specific fragments of the CDL standard (Figure S4). Despite the complexity of CDL structures, we were thus able to confidently pinpoint double bonds and cyclopropane modifications in the different isomers present.

## Conclusions

The impact of lipid interactions across different scales on the structure and function of proteins is difficult to disentangle. Native MS allows us to investigate protein–lipid complexes containing endogenous lipids that remain bound after protein purification or that are selected for binding *in vitro*. Here we introduced a workflow that combines native MS with highly informative lipid fragmentation to gain deep structural information about protein-bound lipids directly from intact protein–lipid complexes. The approach involves multiple stages of isolation and collision-based activation combined with UVPD. We showed that UVPD of released lipids allows us to pinpoint C=C bonds and cyclopropane modifications in protein-bound phospholipids, either from endogenous sources or after incubation with a lipid extract.

A similar approach used to study lipids that copurify with proteins is based on lipid extraction from a purified protein sample followed by LC-MS/MS-based lipidomics.^14^ This method reliably identifies lipids that are enriched in the immediate protein environment by comparison with the bulk lipidome, though not at the isomer level. The protein amounts required are similar to what we need for a complete isomer-resolved analysis of lipids copurified with proteins. However, abundant membrane lipids that are not necessarily bound to the protein are usually pulled down as well. The major advantage of our approach is that we perform a gas-phase purification by *m*/*z* selection of intact protein–lipid complexes. As a consequence, background lipids that do not bind to the protein are eliminated, and we thus ensure that lipids included in the downstream analysis bind either to the protein surface or specific lipid binding sites. Direct infusion further allows us to work on the time scale required for sufficient averaging of UVPD spectra, which would not be possible on the LC-MS/MS time scale of a classical lipidomics analysis.

A disadvantage of the technique is that a relatively high extent of bound lipids must be present initially. For membrane proteins devoid of lipids after detergent purification, this issue can be circumvented by incubation with a lipid extract of the expression host, to screen for lipid binding preferences. Another issue arises since we fragment intact phospholipids containing two to four acyl chains such that assignments can be ambiguous in cases where more than one acyl chain is modified. However, this issue can be alleviated by considering known biochemical pathways of the organism of interest.

The workflow presented here requires no sample or instrument modification. We envision that it can be extended to characterize more complex protein–lipid interactions in eukaryotic cells under different metabolic conditions, including differences between healthy and cancerous cells. Metabolic changes are known to change lipid isomer composition, ultimately impacting protein function, either by changing the bulk membrane properties or altering specific protein–lipid interactions. The tools presented here provide a means to go beyond detecting cell-wide changes in the lipidome but instead study individual protein–lipid complexes.

## Supplementary Material



## Data Availability

Raw files of mass spectra shown in the manuscript and Supporting Information have been deposited on Figshare (DOI: 10.25446/oxford.29355752). Code for analysis of lipid fragmentation spectra is available at https://github.com/kanalstrahlen/LipoBoundID-UVPD, and a model data set is available on Figshare (DOI: 10.25446/oxford.29355752).

## Notes

Tables of investigated proteins and lipid standards, figures showing all fragment spectra and software-supported lipid assignment, and tables containing UVPD fragment assignments (PDF)
