# Supplementary material for: Deep Structural Characterization of Protein-Bound Lipids via Native MS and Ultraviolet Photodissociation
Source: Anal Chem. 2025 Aug 28;97(35):19331–9. doi: 10.1021/acs.analchem.5c03691 (PMC12424025; doi:10.1021/acs.analchem.5c03691)
Supplement: Supplementary file 1 [file ac5c03691_si_001.pdf]

Supporting Information for:

Deep Structural Characterization of Protein-Bound Lipids via Native MS and Ultraviolet Photodissociation

Carla Kirschbaum,[1,2] Jack L. Bennett,[1,2] and Carol V. Robinson[1,2]*

1. Kavli Institute for Nanoscience Discovery, University of Oxford, Oxford OX1 3QU, U.K.

2. Department of Chemistry, University of Oxford, Oxford OX1 3QU, U.K.

*Correspondence to: carol.robinson@chem.ox.ac.uk

Contents

Overview of investigated proteins and lipid standards S3

Table S1. ..... S3

Table S2. ..... S3

UVPD spectra of lipid standards S4

Figure S1 ..... S4

Figure S2 ..... S5

Figure S3 ..... S6

Figure S4 ..... S6

Table S3. ..... S7

Figure S5 ..... S8

Software-supported assignment of lipid structures S9

Figure S6 ..... S9

Fragment spectra of lipids bound to MlaC S10

Figure S7 ..... S10

Figure S8 ..... S11

Table S4. ..... S12

Figure S9 ..... S13

Table S5. ..... S14

Fragment spectra of lipids bound to AqpZ S17

Figure S10 ..... S17

Figure S11. ..... S18

Figure S12 ..... S19

Overview of investigated proteins and lipid standards

Table S1. Expected and measured masses of proteins investigated in this work.

Protein	Expected mass (kDa)	Measured mass (KDa)
MlaC	21.790	21.790
AqpZ (tetramer)	98.565	98.918

Table S2. Lipid standards investigated in this work.

POPC IsoPure<br>PC 16:0/18:1(9Z)	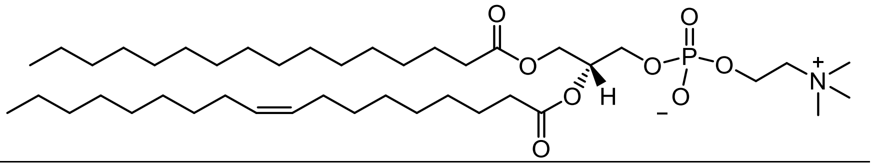
OPPC IsoPure<br>PC 18:1(9Z)/16:0	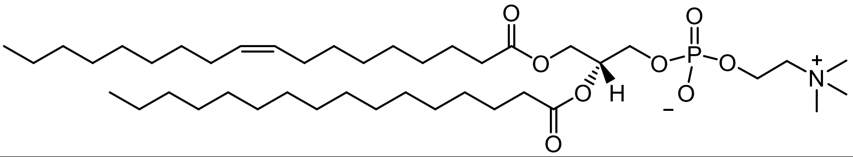
DOPG<br>PG 18:1(9Z)/18:1(9Z)	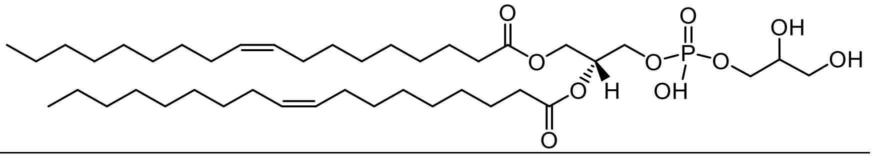
DOPE<br>PE 18:1(9Z)/18:1(9Z)	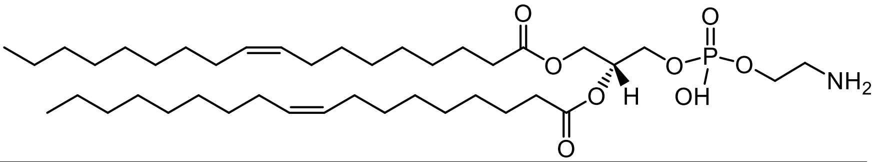
PE 16:0/17:1(cy9)	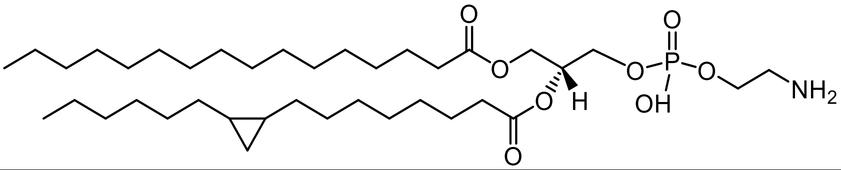
CDL all 18:1(9Z)	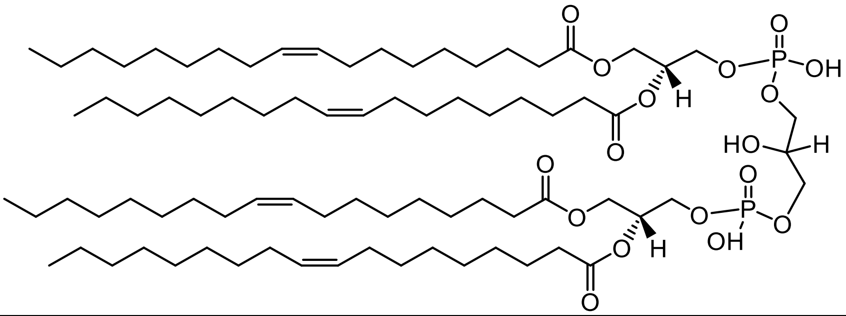

UVPD spectra of lipid standards

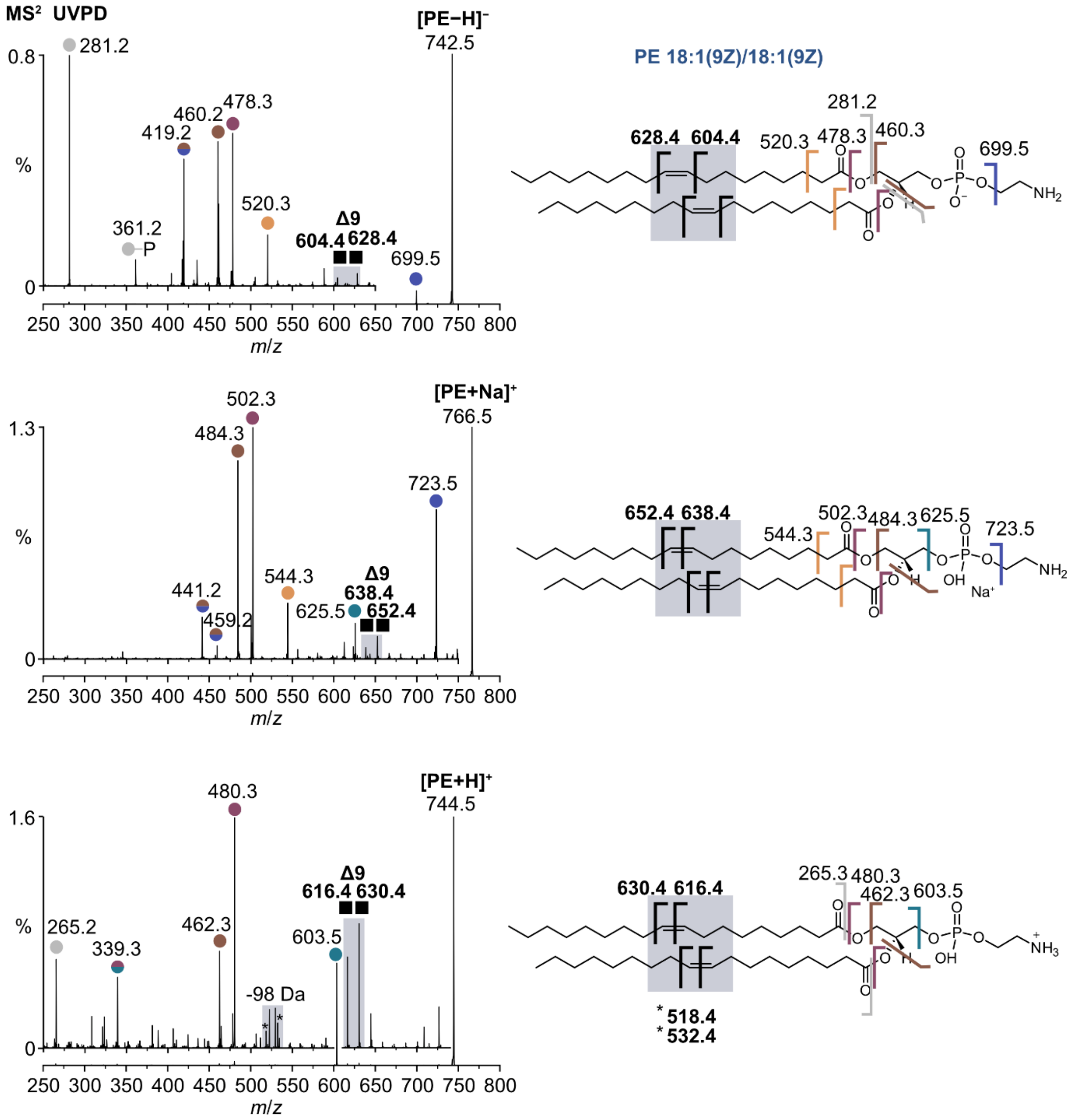

Figure S1. UVPD spectra of deprotonated, sodiated and protonated DOPE with assigned fragments (P = phosphate). In positive ion mode, double bonds yield fragment ion pairs spaced by 14 instead of 24 Da. Furthermore, protonated DOPE yields a fragment ion pair spaced by 14 Da which is shifted by −98 Da relative to the diagnostic double bond fragments (*).

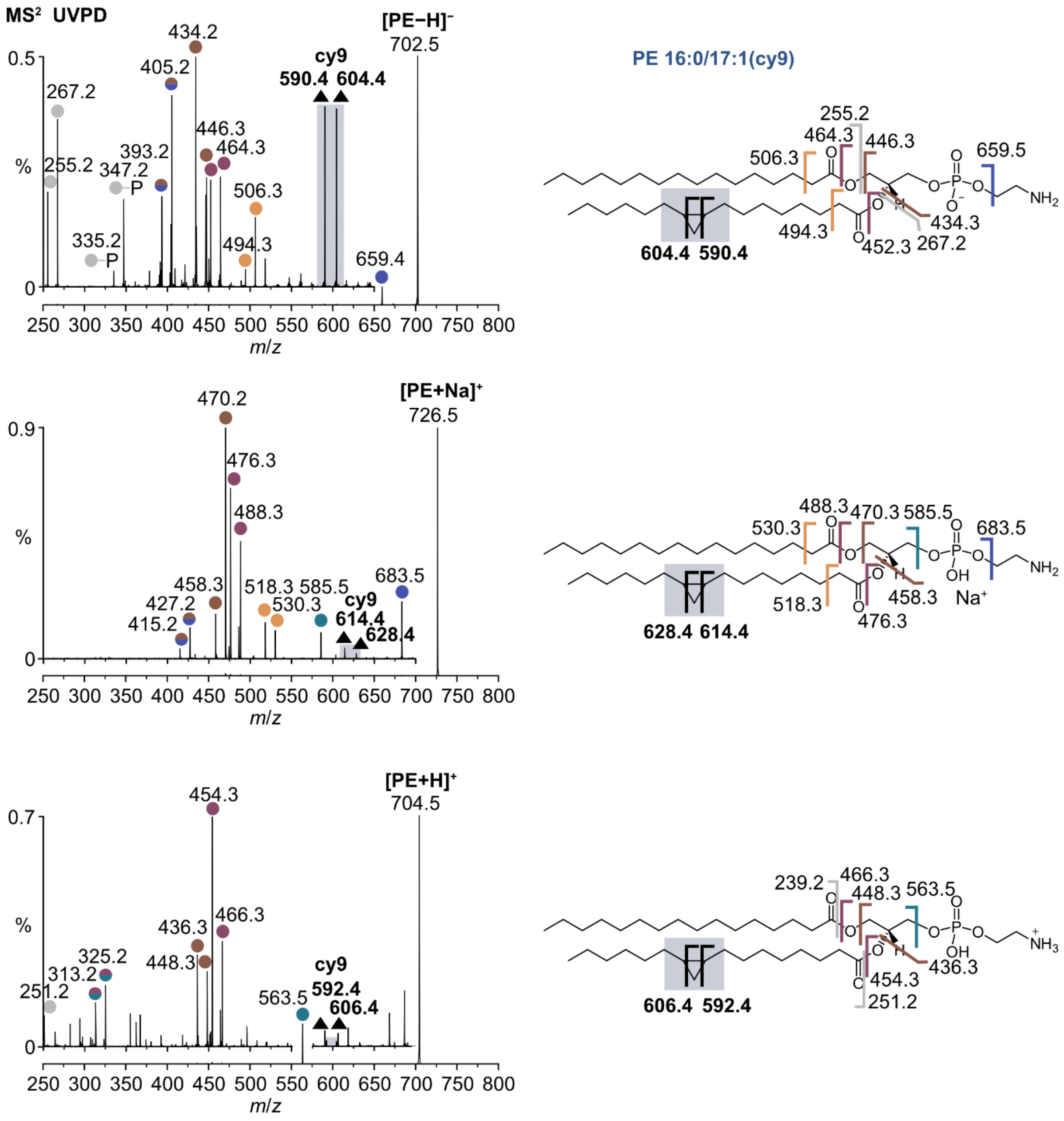

Figure S2. UVPD spectra of deprotonated, sodiated and protonated PE 16:0/17:1(cy9) with assigned fragments (P = phosphate).

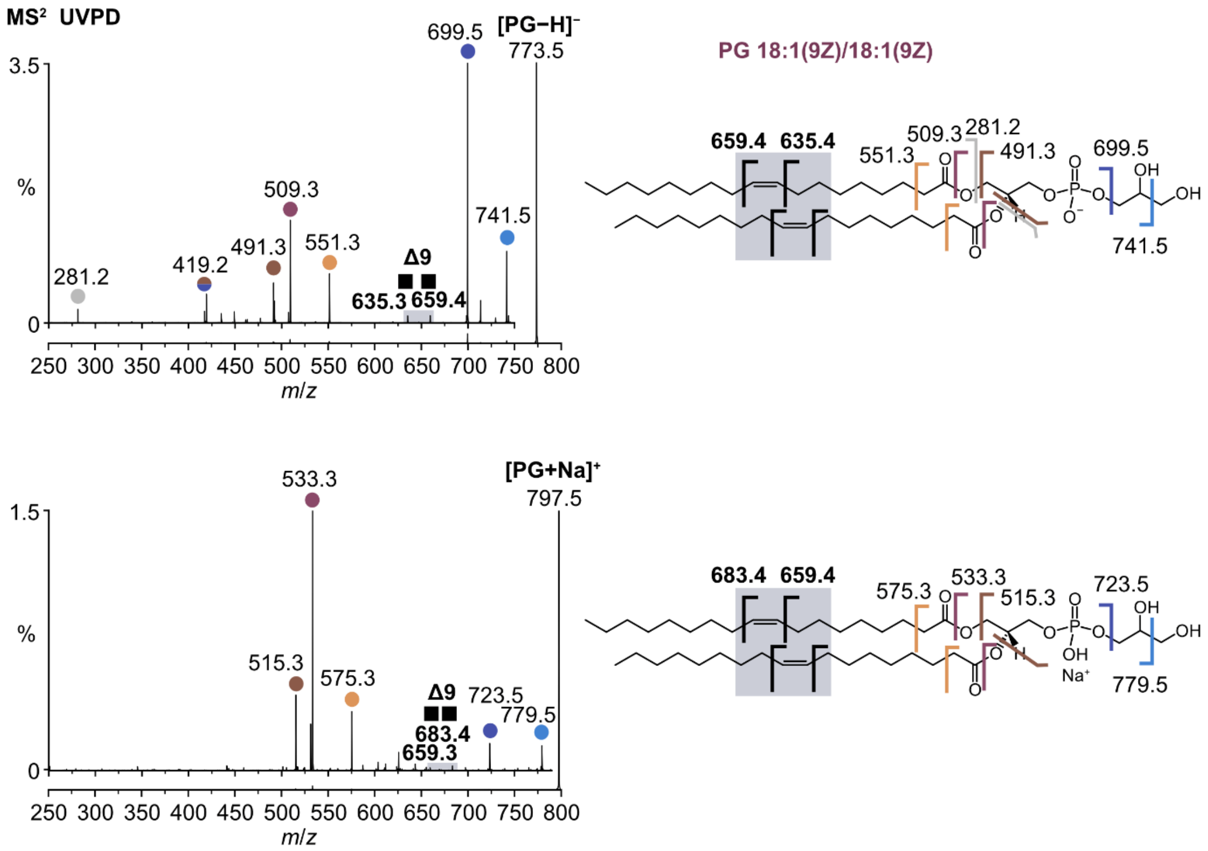

Figure S3. UVPD spectra of deprotonated and sodiated DOPG with assigned fragments.

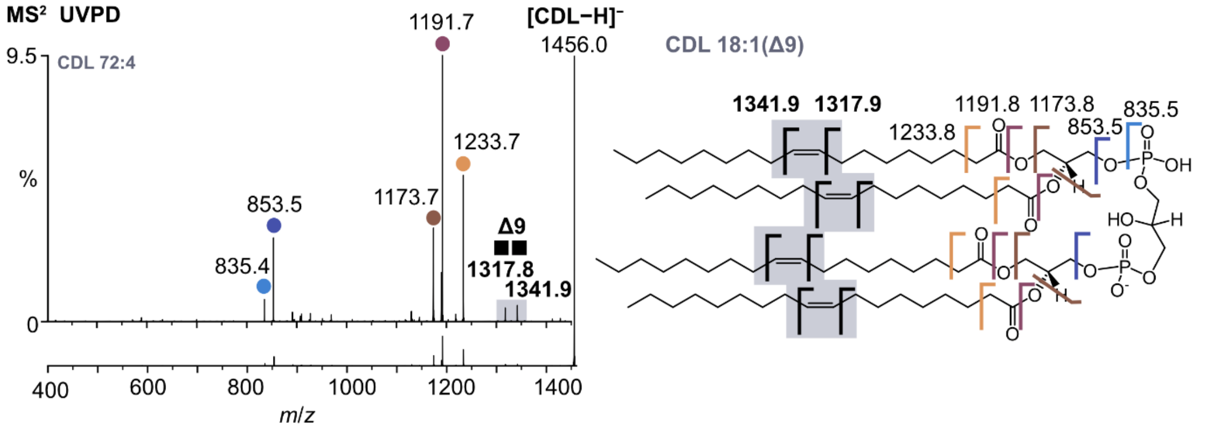

Figure S4. UVPD spectrum and structure of deprotonated cardiolipin CDL (all 18:1(9*Z*)) with assigned fragments.

Table S3. Optimal ion polarity and ion type for the localization of acyl chains (*sn*-position), C=C bonds and cyclopropane modifications in PE, PG and CDL.

Lipid class	Feature	Optimal polarity	Ion type	Fragment spacing
**PE**	C=C bonds	positive	protonated	14 Da
	cyclopropane	negative	deprotonated	14 Da
	sn-position	positive	sodium adduct	-
**PG**	C=C bonds	negative	deprotonated	24 Da
	cyclopropane	negative	deprotonated	14 Da
	sn-position	positive	sodium adduct	-
**CDL**	C=C bonds	negative	deprotonated	24 Da
	cyclopropane	negative	deprotonated	14 Da

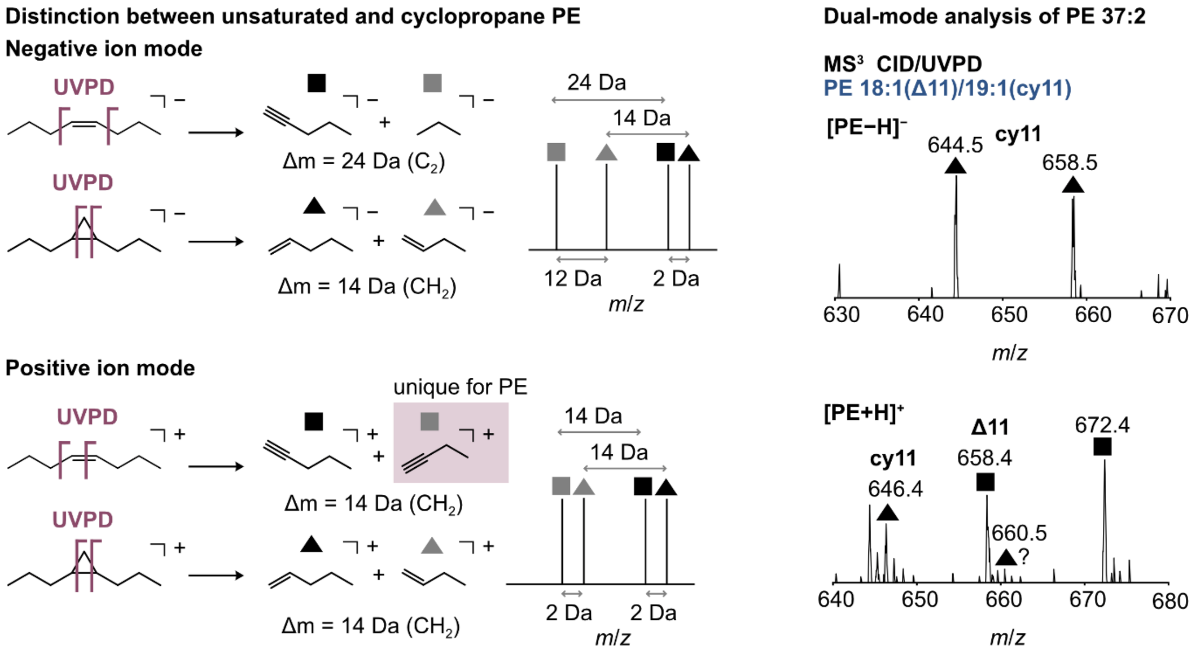

Figure S5. Localization of C=C bonds and cyclopropane in PE lipids. Left: Fragment ion pairs indicating the position of C=C bonds or cyclopropane in PE can be distinguished from each other based on a 2 Da mass shift despite identical spacing (14 Da) for both modifications in positive ion mode. Right: PE lipids containing both modifications are comprehensively analyzed by UVPD in both ion polarities. Here, UVPD spectra of PE 37:2 released from MlaC are shown. Cyclopropane fragments cannot be confidently assigned in positive ion mode but are the predominant species in negative ion mode.

Software-supported assignment of lipid structures

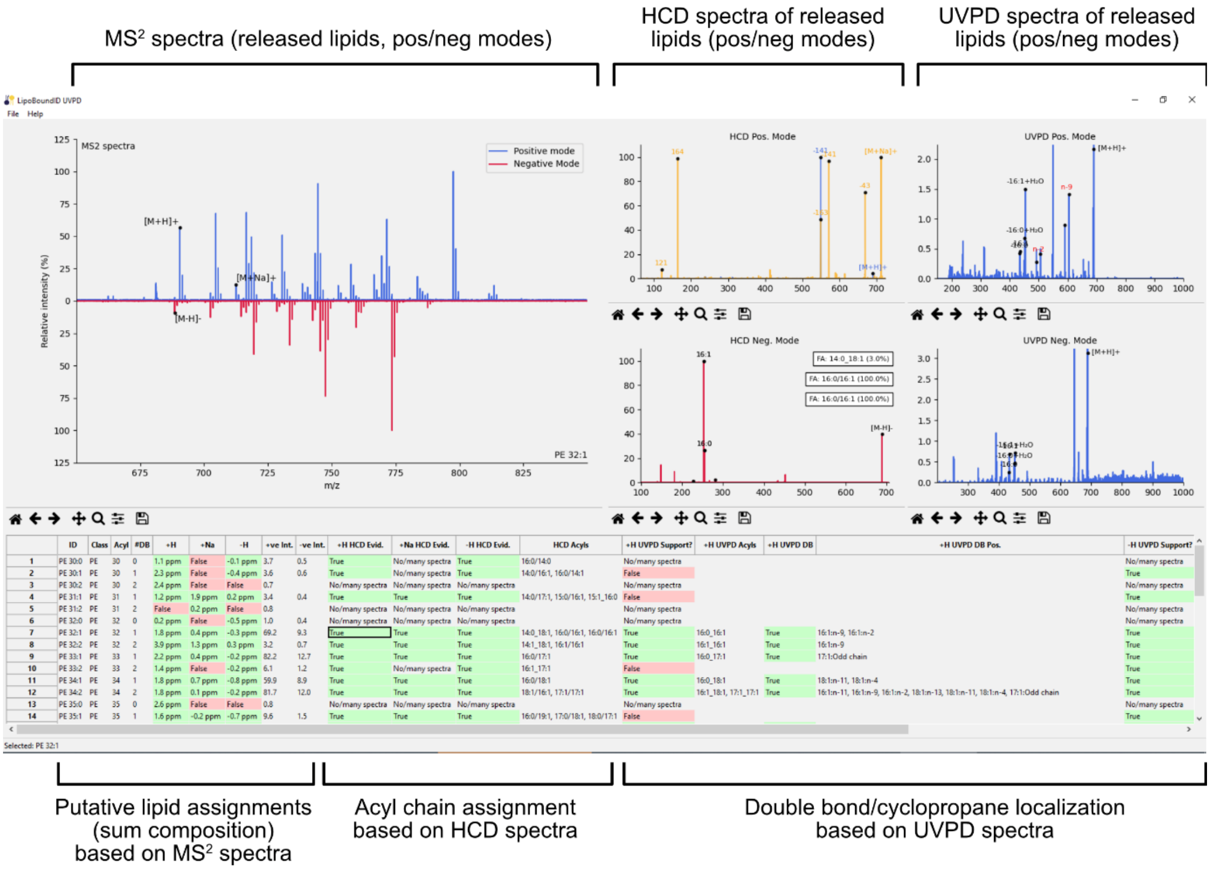

Figure S6. Visual interface of the algorithm used for identification of lipids released from protein–lipid complexes. Putative lipid assignments (sum composition) are made based on exact masses in the high-resolution MS^2 spectra. The lipid class and adduct type are confirmed by positive-mode HCD of individual lipids, and acyl chains are identified by negative-mode HCD. Chain modifications (C=C bonds and cyclopropane) are identified and localized based on UVPD spectra. The assignments are summarized in table format.

Fragment spectra of lipids bound to MlaC

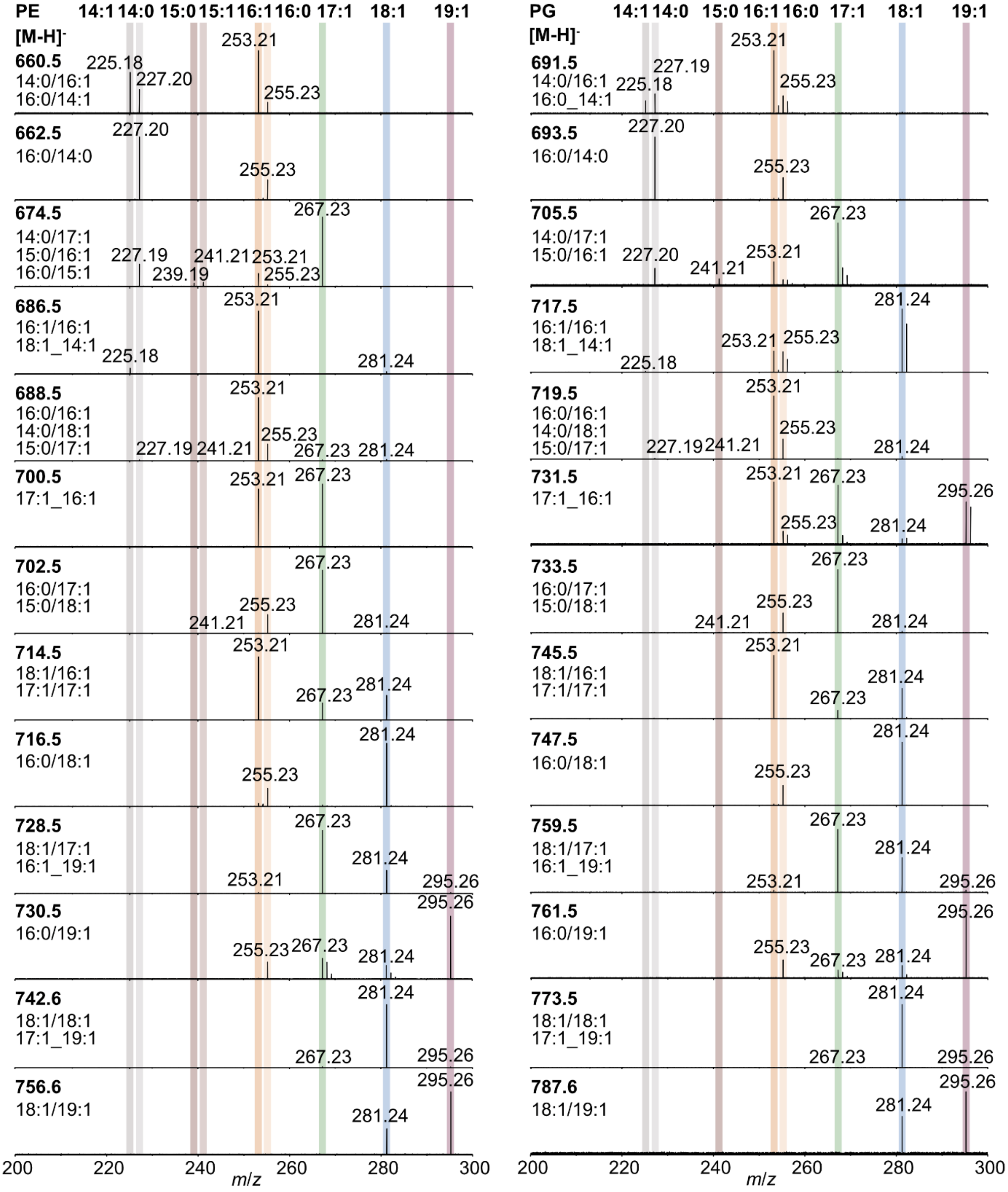

Figure S7. Negative-mode CID/HCD MS^3 spectra of PE and PG lipids released from MlaC reveal the fatty acid composition of each phospholipid. Monoisotopic peaks were isolated to allow detection of overlapping species. Monoisotopic masses of the intact deprotonated phospholipids are indicated together with the identified fatty acid compositions.

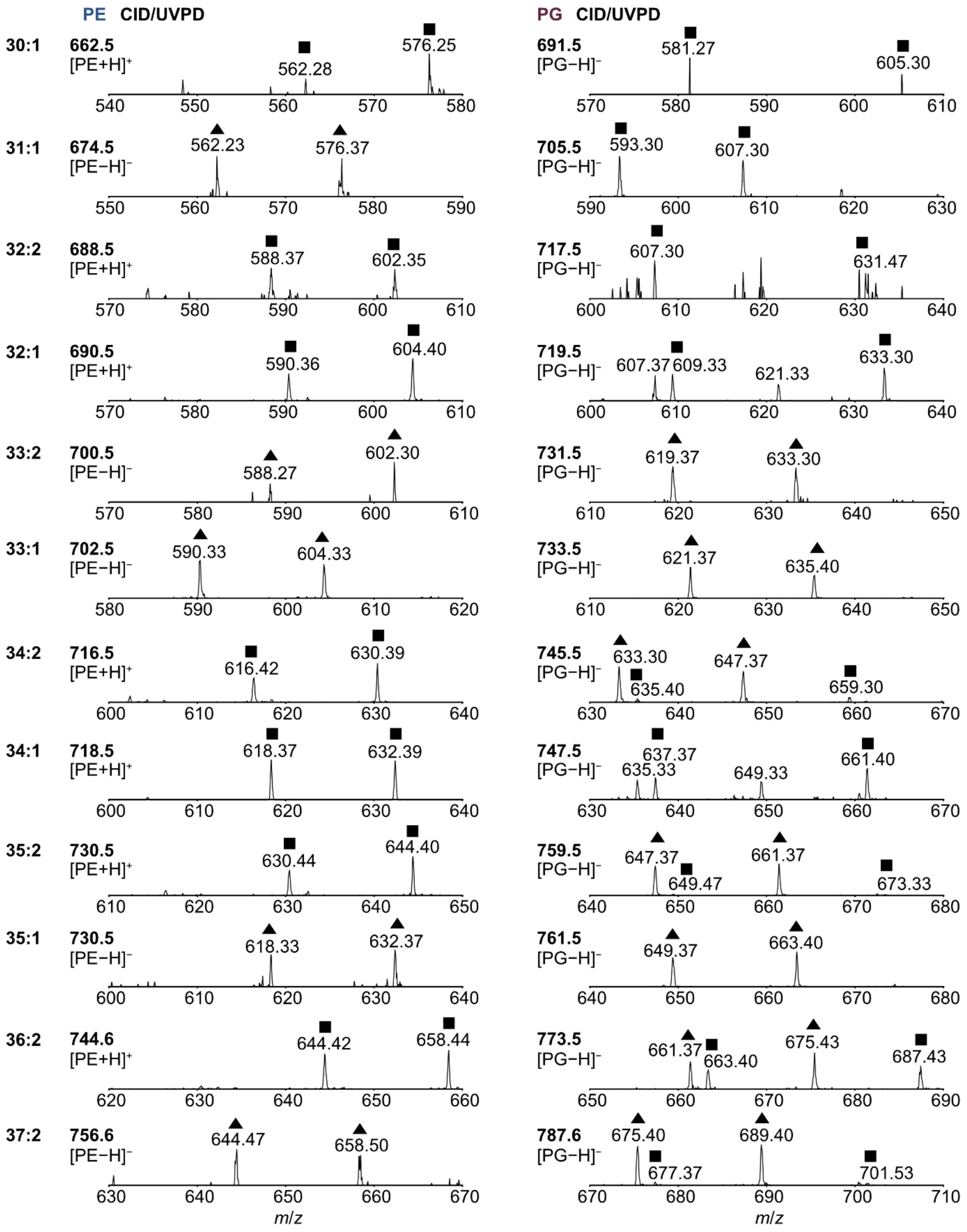

Figure S8. CID/UVPD MS^3 spectra of PE and PG lipids released from MlaC reveal the positions of double bonds and cyclopropane rings. For PEs, the detection of double bonds is enhanced for protonated ions, while cyclopropane lipids are more readily detected in negative ion mode. For PG, both modification types yield fragment ion pairs upon UVPD of deprotonated ions.

Table S4. Fragment masses corresponding with UVPD-induced cross-ring fragmentation of acyl chains depending on their *sn*-position

Fatty acid	*sn*-1	*sn*-2
14:0	291<br>307	249
14:1	289<br>305	247
15:0	305<br>321	263
15:1	303<br>319	261
16:0	319<br>335	277
16:1	317<br>333	275
17:1	331<br>347	289
18:1	345<br>361	303
19:1	359<br>375	317

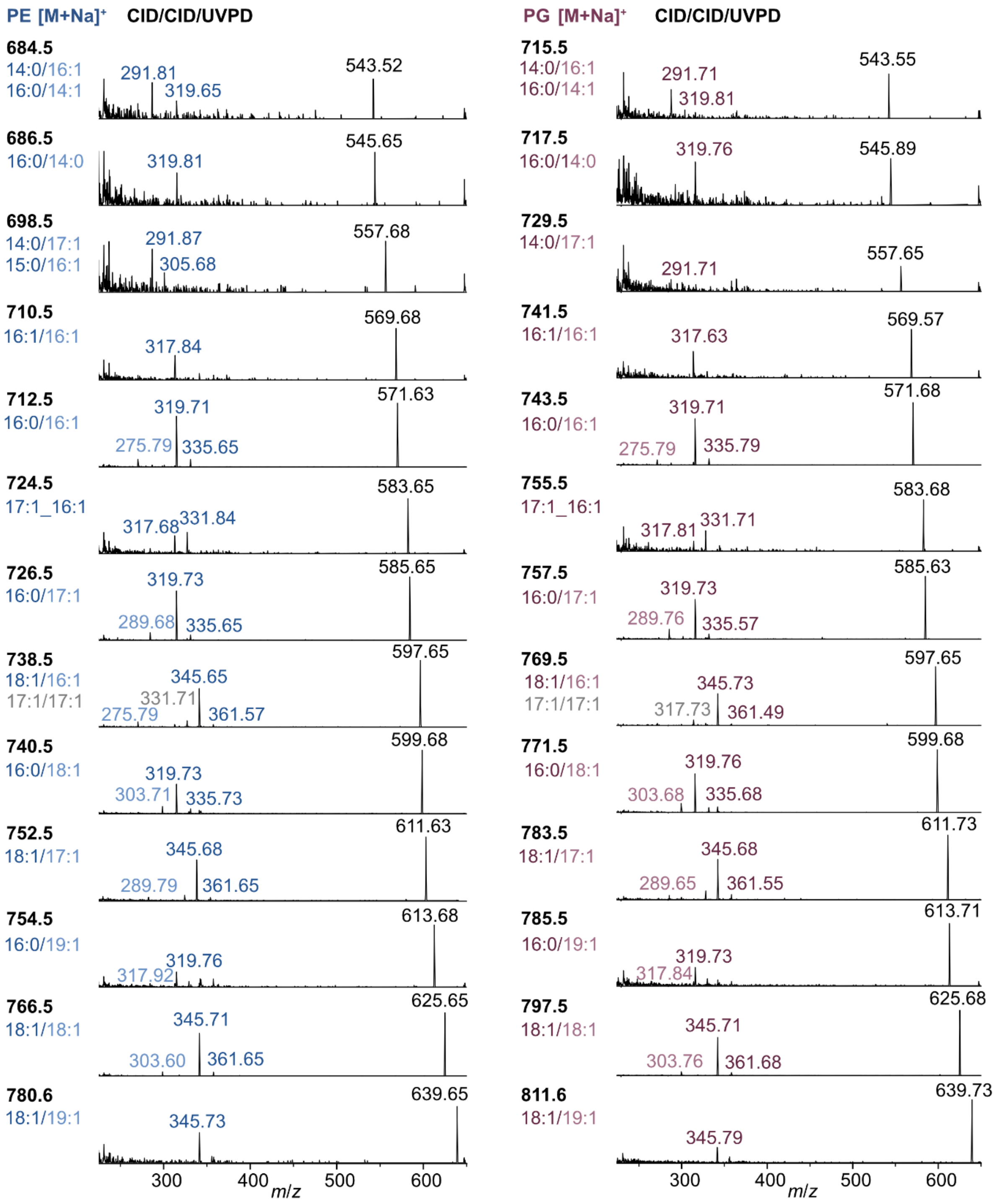

Figure S9. CID/CID/UVPD MS^4 spectra of PE and PG lipids released from MlaC reveal the *sn*-position of acyl chains on the glycerol backbone. Lipids were released using CID in positive ion mode, and sodiated phospholipids were further fragmented using CID. The main fragment resulting from neutral loss of the headgroup was isolated and fragmented by UVPD. Cross-ring fragments contain fatty acyls at the *sn*-1 (dark) or *sn*-2 (light) position (*cf.* Table S4 for fragment assignment).

Table S5. Overview of lipids bound to MlaC, including fatty acid identities, fatty acid positions, and masses of UVPD fragment ions that reveal double bond and cyclopropane modifications.

Lipid	*m/z*	pos	neg	Fatty acids	pos H$^+$	pos Na$^+$	neg
**PE 30:1**	M+H$^+$<br>M+Na$^+$	662.5<br>684.5	660.5	14:0/16:1<br>16:0/14:1	**Δ9/7**<br>562.3,<br>576.3	**Δ9/7**<br>584.3,<br>598.4	**Δ9/7**<br>550.4,<br>574.3
**PE 30:0**	M+H$^+$<br>M+Na$^+$	664.5<br>686.5	662.5	16:0/14:0	n.a.	n.a.	n.a.
**PE 31:1**	M+H$^+$<br>M+Na$^+$	676.5<br>698.5	674.5	14:0/17:1<br>15:0/16:1<br>16:0/15:1	-	**Cy9/7**<br>586.4,<br>600.4	**Cy9/7**<br>562.2,<br>576.4
**PE 32:2**	M+H$^+$<br>M+Na$^+$	688.5<br>710.5	686.5	16:1/16:1<br>18:1_14:1	**Δ9**<br>588.4,<br>602.4	**Δ9**<br>610.4,<br>624.3	-
**PE 32:1**	M+H$^+$<br>M+Na$^+$	690.5<br>712.5	688.5	16:0/16:1<br>14:0/18:1<br>15:0/17:1	**Δ9**<br>590.4,<br>604.4	**Δ9**<br>612.3,<br>626.4	-
**PE 33:2**	M+H$^+$<br>M+Na$^+$	702.5<br>724.5	700.5	17:1_16:1<br>Isomer mix	**Δ9/11**<br>602.3,<br>616.3<br>**Cy9/7**<br>590.3,<br>604.5	**Δ9/11**<br>624.3,<br>638.4<br>**Cy9/7**<br>612.3,<br>626.5	**Cy9/7**<br>588.3,<br>602.3
**PE 33:1**	M+H$^+$<br>M+Na$^+$	704.5<br>726.5	702.5	16:0/17:1<br>15:0/18:1	-	**Cy9**<br>614.4,<br>628.4	**Cy9**<br>590.3,<br>604.3
**PE 34:2**	M+H$^+$<br>M+Na$^+$	716.5<br>738.5	714.5	18:1/16:1<br>17:1/17:1	**Δ9/11**<br>616.4,<br>630.4<br>**Cy9**<br>604.3,<br>618.4	**Δ9/11**<br>638.3,<br>652.4<br>**Cy9**<br>626.4,<br>640.3	**Cy9**<br>602.3,<br>616.4
**PE 34:1**	M+H$^+$<br>M+Na$^+$	718.5<br>740.5	716.5	16:0/18:1	**Δ11**<br>618.4,<br>632.4	**Δ11**<br>640.4,<br>654.4	-
**PE 35:2**	M+H$^+$<br>M+Na$^+$	730.5<br>752.5	728.5	18:1/17:1<br>16:1_19:1<br>Isomer mix	**Δ11/Δ9**<br>630.4,<br>644.4<br>**Cy9**<br>618.4,<br>632.5	**Δ11/Δ9**<br>652.4,<br>666.4<br>**Cy9**<br>640.3,<br>654.4	**Δ11/Δ9**<br>618.4,<br>642.3<br>**Cy9**<br>616.4,<br>630.4
**PE 35:1**	M+H$^+$<br>M+Na$^+$	732.5<br>754.5	730.5	16:0/19:1	-	**Cy11**<br>642.41,<br>656.426	**Cy11**<br>618.3,<br>632.4

**PE 36:2**	M+H⁺<br>M+Na⁺	744.6<br>766.5	742.6	18:1/18:1<br>17:1_19:1<br>Isomer mix	**Δ11**<br>644.4,<br>658.4	**Δ11**<br>666.4,<br>680.4<br>**Cy9/11**<br>654.4,<br>668.5	**Δ11**<br>632.5,<br>656.4<br>Cy9/11<br>630.3,<br>644.3
**PE 37:2**	M+H⁺<br>M+Na⁺	758.6<br>780.6	756.6	18:1/19:1	**Δ11**<br>658.4,<br>672.4<br>**Cy11**<br>646.4,<br>660.5	**Δ11**<br>680.4,<br>694.4<br>**Cy11**<br>668.3,<br>682.4	**Cy11**<br>644.5,<br>658.5
**PG 30:1**	M+Na⁺	715.5	691.5	14:0/16:1<br>16:0/14:1	n.a.	**Δ9**<br>605.2,<br>629.3	**Δ9**<br>581.3,<br>605.3
**PG 30:0**	M+Na⁺	717.5	693.5	16:0/14:0	n.a.	n.a.	n.a.
**PG 31:1**	M+Na⁺	729.5	705.5	14:0/17:1<br>15:0/16:1	n.a.	**Cy9**<br>617.3,<br>631.3	**Cy9**<br>593.3,<br>607.3
**PG 32:2**	M+Na⁺	741.5	717.5	16:1/16:1<br>18:1_14:1	n.a.	**Δ9**<br>631.4,<br>655.4	**Δ9**<br>607.3,<br>631.2
**PG 32:1**	M+Na⁺	743.5	719.5	16:0/16:1<br>14:0/18:1<br>15:0/17:1	n.a.	**Δ9**<br>633.5,<br>657.3	**Δ9**<br>609.3,<br>633.3<br>**Cy9**<br>607.4<br>621.3
**PG 33:2**	M+Na⁺	755.5	731.5	17:1_16:1<br>isomer mix	n.a.	**Δ9**<br>645.4,<br>669.4<br>**Cy9**<br>643.4,<br>657.3	**Δ9**<br>621.4,<br>645.4<br>**Cy9**<br>619.4,<br>633.3
**PG 33:1**	M+Na⁺	757.5	733.5	16:0/17:1<br>15:0/18:1	n.a.	**Cy9**<br>645.3,<br>659.4	**Cy9**<br>621.4,<br>635.4
**PG 34:2**	M+Na⁺	769.5	745.5	18:1/16:1<br>17:1/17:1	n.a.	**Δ11/9**<br>659.4,<br>683.4<br>**Cy9**<br>657.3,<br>671.4	**Δ11/9**<br>635.4,<br>659.3<br>**Cy9**<br>633.4,<br>647.4

**PG 34:1**	M+Na⁺	771.5	747.5	16:0/18:1	n.a.	**Δ11**<br>661.4,<br>685.5	**Δ11**<br>637.4,<br>661.4
**PG 35:2**	M+Na⁺	783.5	759.5	18:1/17:1<br>16:1_19:1<br>Isomer mix	n.a.	**Δ11**<br>673.3,<br>697.4<br>**Cy9/11**<br>671.3,<br>685.3	**Δ11**<br>649.5,<br>673.3<br>**Cy9/Cy11**<br>647.4,<br>661.4
**PG 35:1**	M+Na⁺	785.5	761.5	16:0/19:1	n.a.	**Cy11**<br>673.4,<br>687.4	**Cy11**<br>649.4,<br>663.4
**PG 36:2**	M+Na⁺	797.5	773.5	18:1/18:1<br>17:1_19:1<br>Isomer mix	n.a.	**Δ11**<br>687.6,<br>711.4	**Δ11**<br>663.4,<br>687.4<br>**Cy9/11**<br>661.4,<br>675.4
**PG 37:2**	M+Na⁺	811.6	787.6	18:1/19:1	n.a.	**Δ11**<br>701.4,<br>725.4<br>**Cy11**<br>699.4,<br>713.5	**Δ11**<br>677.4,<br>701.5<br>**Cy11**<br>675.4,<br>689.4

Fragment spectra of lipids bound to AqpZ

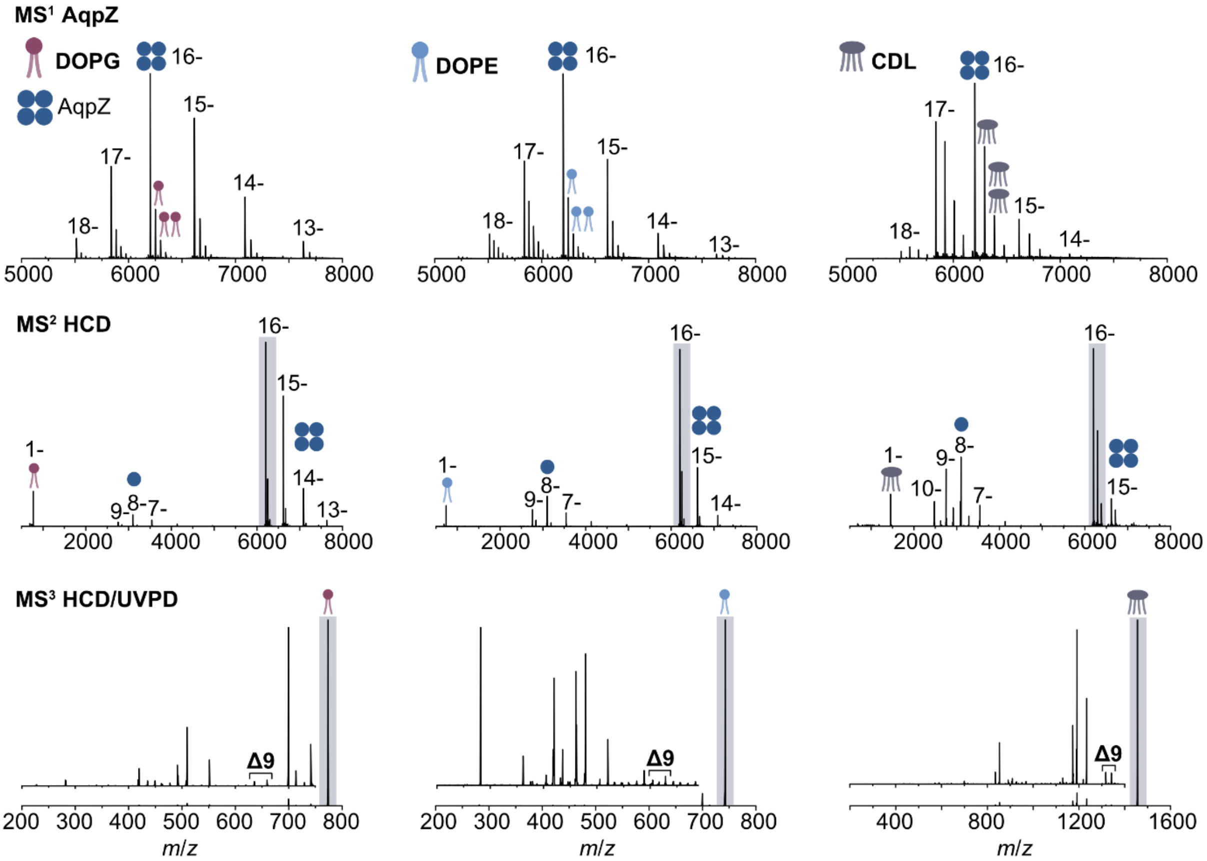

Figure S10. Binding, release and characterization of lipid standards incubated with aquaporin Z (AqpZ). DOPG, DOPE and CDL (18:1) bind to AqpZ in solution and are released as singly charged anions upon HCD in negative ion mode. UVPD of released lipids allows to define double bond positions (all Δ9).

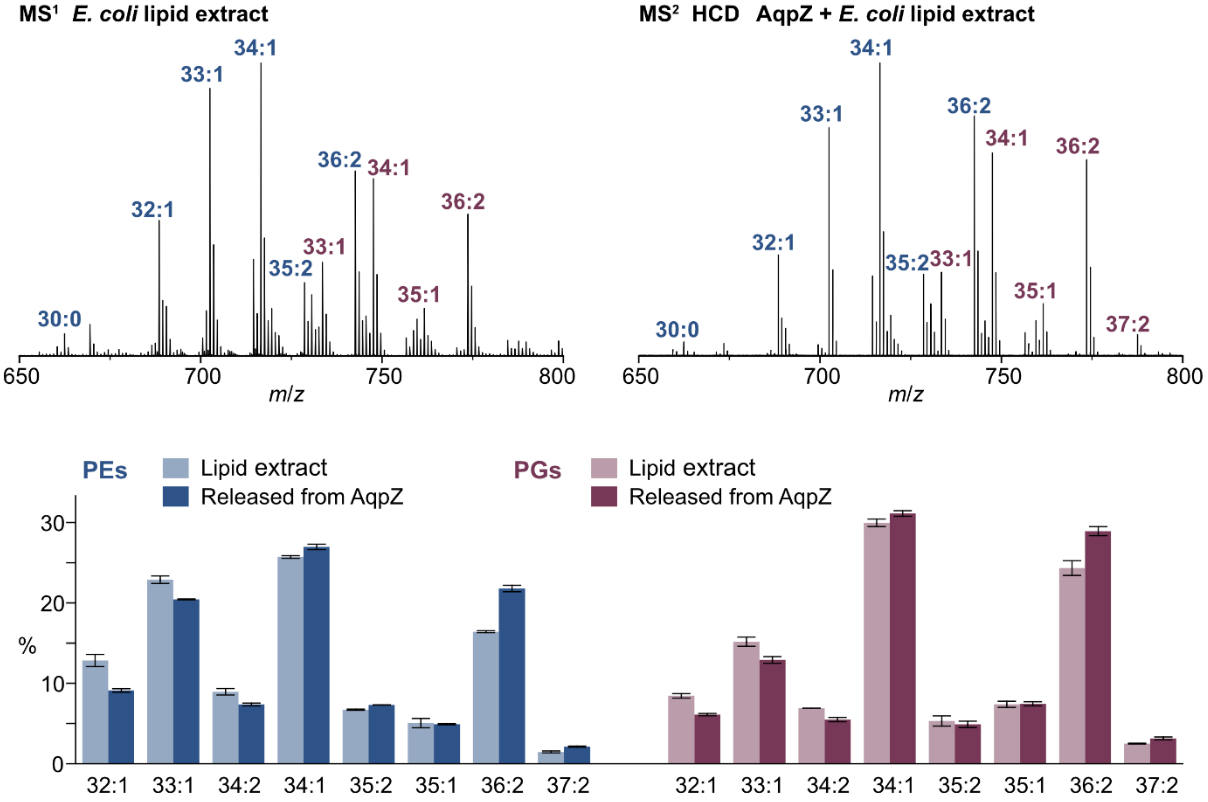

Figure S11. *E. coli* lipids binding to AqpZ. The profile of phospholipids binding to AqpZ does not differ significantly from the lipid extract, suggesting little lipid specificity of AqpZ. Left: mass spectrum of *E. coli* lipid extract (negative mode). Right: Phospholipids released from AqpZ using HCD in negative ion mode after incubation with the *E. coli* lipid extract. Phospholipids with longer lipid chains (>34) are slightly enriched in the lipid ensemble released from AqpZ.

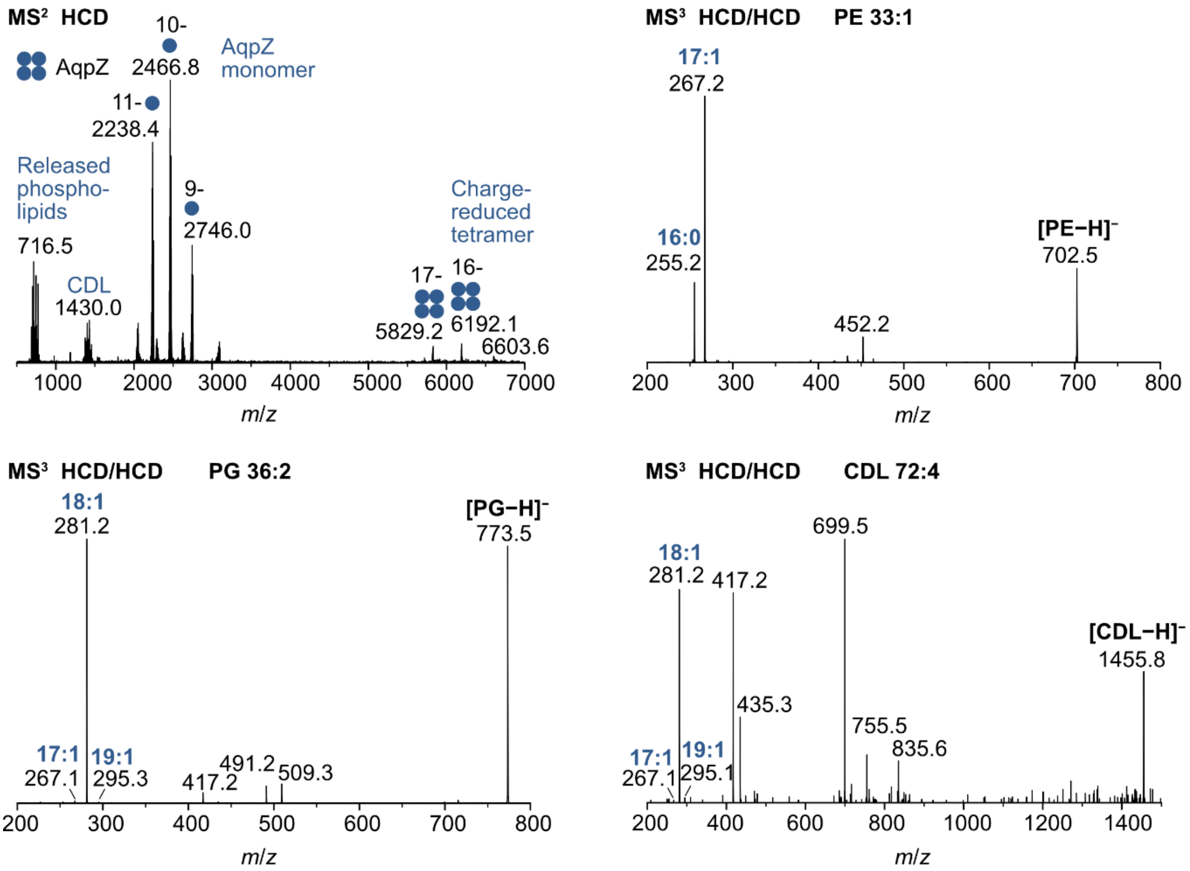

Figure S12. Fatty acyl determination of *E. coli* lipids released from AqpZ. Collisional activation of AqpZ–lipid complexes (here charge state 17-) leads to release of singly charged lipids and charge reduction of the protein. Release of cardiolipins requires elevated collision energies, leading to substantial protein dissociation into monomers. MS^3 (HCD/HCD) spectra are shown for PE 33:1, PG 36:2, and CDL 72:4 with fatty acid carboxylates annotated.
